# Hypergraph-based analysis of weighted gene co-expression hypernetwork

**DOI:** 10.3389/fgene.2025.1560841

**Published:** 2025-04-04

**Authors:** Libing Bai, Zongjin Li, Chunyang Tang, Changxin Song, Feng Hu

**Affiliations:** ^1^ Computer College of Qinghai Normal University, Xining, Qinghai, China; ^2^ The State Key Laboratory of Tibetan Intelligence, Qinghai, Xining, China; ^3^ College of Science, North China University of Science and Technology, Tangshan, China; ^4^ Department of Mechanical Engineering and Information, Shanghai Urban Construction Vocational College, Shanghai, China

**Keywords:** hypergraph, higher order network, weighted gene co-expression network analysis, gene expression profiling analysis, hierarchical clustering

## Abstract

**Background:**

With the rapid advancement of gene sequencing technologies, Traditional weighted gene co-expression network analysis (WGCNA), which relies on pairwise gene relationships, struggles to capture higher-order interactions and exhibits low computational efficiency when handling large, complex datasets.

**Methods:**

To overcome these challenges, we propose a novel Weighted Gene Co-expression Hypernetwork Analysis (WGCHNA) based on weighted hypergraph, where genes are modeled as nodes and samples as hyperedges. By calculating the hypergraph Laplacian matrix, WGCHNA generates a topological overlap matrix for module identification through hierarchical clustering.

**Results:**

Results on four gene expression datasets show that WGCHNA outperforms WGCNA in module identification and functional enrichment. WGCHNA identifies biologically relevant modules with greater complexity, particularly in processes like neuronal energy metabolism linked to Alzheimer’s disease. Additionally, functional enrichment analysis uncovers more comprehensive pathway hierarchies, revealing potential regulatory relationships and novel targets.

**Conclusion:**

WGCHNA effectively addresses WGCNA’s limitations, providing superior accuracy in detecting gene modules and deeper insights for disease research, making it a powerful tool for analyzing complex biological systems.

## 1 Introduction

With the rapid advancement of gene sequencing technologies, biological research has entered the era of big data, leading to a swift increase in the variety and scale of gene expression data (Soon et al., 2013). However, extracting meaningful biological information from these complex and voluminous gene datasets has become a significant challenge in current research (Zhang et al., 2024a; Deng et al., 2023). In light of the dramatic growth of data and the intricate interactions among genes, many traditional bioinformatics analysis methods (such as statistical correlation-based approaches) are gradually proving inadequate for modern research demands (Del Val et al., 2024). Consequently, researchers have introduced Weighted Gene Co-Expression Network Analysis (WGCNA), which has become a powerful tool for analysing gene co-expression patterns (Langfelder and Horvath, 2008; Liang et al., 2018; Liu et al., 2016; Soleimani Zakeri et al., 2020). WGCNA not only identifies modules of co-expressed genes and reveals their associations with biological traits but also aids researchers in better understanding the underlying mechanisms of gene networks, facilitating the discovery of potential disease biomarkers and therapeutic targets (Jha et al., 2024). This method has achieved significant results in the study of various diseases, providing new insights for precision medicine and disease treatment (Abudereheman et al., 2024; Chen et al., 2024).

Although WGCNA has shown remarkable performance in constructing gene co-expression networks, existing improvements have primarily focused on aspects such as node similarity metrics (Zhang and Wong, 2022; Hou et al., 2021), clustering algorithms (Botía et al., 2017; Greenfest-Allen et al., 2017), and data compatibility (Zoppi et al., 2021; Ke and Ge, 2024). For instance, numerous studies have aimed to enhance the accuracy of network construction by optimizing weighted correlation coefficients or distance functions (Hou et al., 2022; Iancu et al., 2015). Concurrently, advancements in clustering algorithms have emerged as a research hotspot, including methods based on modularity measures and dynamic tree cutting (Melo et al., 2024; Yu et al., 2023), which aim to improve the accuracy of module detection. Moreover, the applicability of WGCNA has expanded, as it now supports the integration of various data types beyond transcriptomics, including proteomics and epigenomics (Ma et al., 2024; Cao et al., 2024; Zhang et al., 2024b; Xu et al., 2024). These improvements have significantly enhanced WGCNA’s flexibility and robustness across multi-dimensional datasets, leading to its widespread application in biomedical research (Demirbaga et al., 2024; Rezaei et al., 2022).

WGCNA primarily characterizes pairwise relationships within gene co-expression networks by constructing co-expression networks through the calculation of similarity between genes. However, this approach fails to adequately capture the more complex higher-order interactions among genes, such as patterns of multi-gene cooperation, thereby imposing certain limitations on the model’s ability to elucidate intricate biological network structures (Feng et al., 2021; Tian et al., 2009; Barton et al., 2023). Furthermore, in large-scale datasets and multi-gene interaction analyses, WGCNA faces challenges regarding computational efficiency and the accuracy of module extraction. Specifically, when handling thousands of genes and their associated interactions, there remains room for further optimization in the model’s computational complexity and the precision of module delineation (Rezaie et al., 2023; Yang et al., 2024).

To address the above problems, this paper proposes a weighted gene co-expression hypernetwork analysis (WGCHNA) based on weighted hypergraph. The algorithm introduces hypergraph theory into gene co-expression network analysis. First, a weighted gene co-expression hypernetwork is constructed for the preprocessed gene expression profiles. Then, the topological overlap matrix is calculated through the hypergraph Laplacian matrix. Finally, the gene co-expression modules are mined using hierarchical clustering. To demonstrate the applicability of the algorithm, we compare it with traditional WGCNA (such as PyWGCNA and R WGCNA) and distance-correlated weighted gene co-expression network analysis (DC-WGCNA) methods, and analyze them from the aspects of scale-free property verification, clustering performance evaluation, and time complexity. Experimental results show that the hypergraph can more accurately capture the complex connection relationship between genes and samples, highlight the pathogenic gene module, and effectively screen out key genes significantly related to the disease, demonstrating its practicality and reliability in biological data analysis.

**FIGURE 1:**
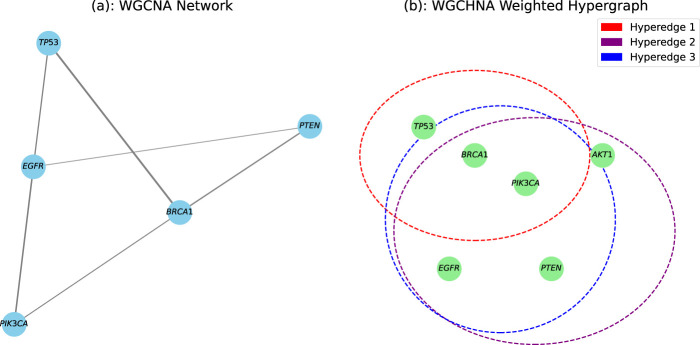
The difference between WGCNA and WGCHNA in the gene interaction network. **(a)** WGCNA Network. **(b)** WGCHNA Weighted Hypergraph.

To more intuitively demonstrate the significant advantages of the WGCHNA method in characterizing high-order gene interactions, we compared the differences between WGCHNA and traditional WGCNA in constructing gene interaction networks (as shown in Figure 1). In the traditional WGCNA shown in Figure 1a, the network nodes represent genes, the edges between nodes represent the co-expression relationship between gene pairs, and the thickness of the edge represents the co-expression strength. This weighted network structure can only capture the direct co-expression relationship between pairs of genes, but it is difficult to reveal more complex multi-gene collaborative interaction patterns. For example, TP53 and BRCA1 may jointly participate in cell cycle regulation, and their direct co-expression relationship can be intuitively represented by a weighted edge, but when more genes are involved in co-regulation, this structure is insufficient. In contrast, as shown in Figure 1b, the WGCHNA method we proposed adopts a weighted hypergraph structure. The nodes in WGCHNA also represent genes, but samples are used as hyperedges. The weight of the hyperedge is aggregated according to the correlation between multiple genes. The thickness of the hyperedge represents the sum of the weights of multiple nodes connected by the hyperedge. This method can not only reflect the direct co-expression relationship between paired genes, but more importantly, it can reveal the complex co-regulatory patterns between multiple genes. For example, TP53, BRCA1, and PIK3CA may be involved in the same cancer signaling pathway. WGCHNA can connect these three genes with hyperedges at the same time, which more intuitively shows their potential co-regulatory effects. Therefore, compared with traditional WGCNA, WGCHNA can more effectively and comprehensively reveal high-order interaction networks in genomics.

## 2 Materials and methods

### 2.1 Datasets

The gene data utilized in this study were sourced from the (MODEL-AD) database, specifically the mouse Alzheimer’s disease dataset (5xFAD) (Forner et al., 2021), as well as the (GEO) database, which includes datasets for breast cancer (GSE48213) (Daemen et al., 2013) and hypertension (GSE75360, GSE75670) (Dluzen et al., 2016). Detailed information for each dataset is presented in Table 1.

**TABLE 1:** Details of experimental datasets.

Datasets	Number of genes	Number of samples
5xFAD	55448	192
GSE48213	36953	56
GSE75360	47231	21
GSE75670	15840	12

### 2.2 Extension of gene co-expression networks based on weighted hypergraphs

WGCNA(5) typically constructs networks based on the correlation between pairs of genes, using an adjacency matrix to represent the direct co-expression relationships between gene pairs. However, WGCNA faces challenges in capturing the global features of the network and the complex higher-order regulatory patterns involved in multi-gene cooperation. To address this limitation, this study proposes a weighted gene co-expression hypernetwork model based on hypergraph theory. In this model, multiple gene nodes are connected through hyperedges, reflecting the complex cooperative expression relationships across samples.

**FIGURE 2:**
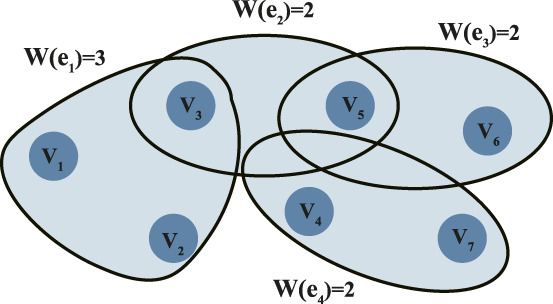
Weighted hypergraph.

Compared to traditional adjacency matrices, the Laplacian matrix of a hypergraph provides a more comprehensive characterization of the network’s global properties, with significant advantages in identifying gene modular structures and multi-gene cooperation. The Laplacian matrix not only reflects the “flow” or “diffusion” between genes but also effectively handles higher-order associations in hypergraphs, enhancing the network’s analytical capabilities to uncover deeper biological functions. Figure 2 presents an example of a weighted hypergraph (Claude, 1973), containing 7 gene nodes and 4 sample hyperedges. The node set is $V=\left\{v_{1},v_{2},v_{3},v_{4},v_{5},v_{6},v_{7}\right\}$ , and the hyperedge set is $E=\left\{e_{1},e_{2},e_{3},e_{4}\right\}$ , where $e_{1}=\left\{v_{1},v_{2},v_{3}\right\}$ , $e_{2}=\left\{v_{3},v_{5}\right\}$ , $e_{3}=\left\{v_{5},v_{6}\right\}$ and $e_{4}=\left\{v_{4},v_{7}\right\}$ . The hyperedge weight set is $W=\left\{3,2,2,2\right\}$ .

By constructing a weighted hypergraph and calculating its Laplacian matrix, we further integrate it into the computation of the Topological Overlap Matrix (TOM). Combining this with a hierarchical clustering algorithm, we can identify key gene modules that reveal the co-expression patterns of genes across different samples. This approach enhances the identification of complex biological modules and improves the analytical capacity of gene co-expression networks.

### 2.3 Weighted gene co-expression hypernetwork analysis

**FIGURE 3:**
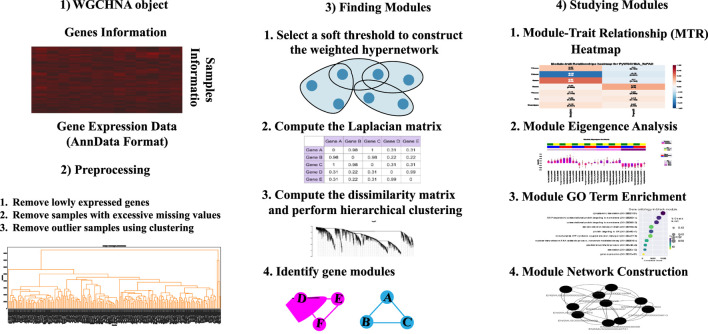
Weighted Gene Co-expression HyperNetwork Analysis (WGCHNA) workflow.

To more precisely capture complex higher-order interactions between genes and improve computational efficiency for large-scale datasets, we designed the algorithmic workflow of Weighted Gene Co-expression HyperNetwork Analysis (WGCHNA), as illustrated in Figure 3. This workflow encompasses data preprocessing, hypergraph construction, gene module identification, and functional enrichment analysis, ultimately revealing the relationships between modules and the potential functions of key genes.

As shown in the Figure 3, WGCHNA constructs a weighted hypergraph that connects multiple genes, capturing the complex patterns of co-expression across samples. The following section provides a detailed description of the specific steps involved in the algorithm.

#### 2.3.1 Construction process and weight definition of weighted hypergraph

##### 2.3.1.1 Hypergraph construction method

     This study constructs a weighted gene co-expression hypernetwork (WGCHNA) based on the gene expression spectrum matrix. Let the gene set V be the node set of the hypergraph, the sample set E be the hyperedge set, and each hyperedge e connects all the genes in the sample. The hyperedge weight matrix W reflects the co-expression strength of gene sets in different samples.

Different from the traditional gene co-expression network, WGCHNA adopts sample-level hyperedge modeling, that is, each sample forms a hyperedge, and all genes in the sample are regarded as a high-order co-expression unit. This design is in line with the idea of hypergraph modeling, because hyperedges not only connect multiple genes, but also connect a set of genes that may be biologically related.

##### 2.3.1.2 Definition of hyperedge weight

     In order to characterize the co-expression strength of gene sets, we define the weight $W_{e}$ of hyperedge e as an aggregate measure of the expression correlation between gene pairs in the sample. The specific calculation is shown in Formula 1:

$$W_{e}=\frac{1}{|P_{e}|}\underset{g_{i},g_{j}\in P_{e}}{\sum }corr\left(g_{i},g_{j}\right)$$ (1)

Among them, $P_{e}$ represents the set of all gene pairs within hyperedge e , $corr\left(g_{i},g_{j}\right)$ is the expression correlation of genes $g_{i}$ and $g_{j}$ in all samples, and $|P_{e}|$ is the total number of gene pairs within hyperedge e . Different from the traditional weighted gene co-expression network (WGCNA) that only builds a network based on the correlation of paired genes, WGCHNA uses sample-level hyperedges in the hypergraph structure to aggregate the expression relationship of multiple genes, thereby improving the model’s ability to capture high-order gene interactions.

Our method follows the core idea of hypergraph modeling, that is, hyperedges are not limited to pairs of genes, but organize multiple genes together to form a higher-order structure. Although the weight calculation uses the aggregation of pairwise correlations, its purpose is to quantify the co-expression pattern of multiple genes in the same sample, rather than simple pairwise interactions. Therefore, this method can capture the higher-order co-expression effects of the entire genome, such as the synchronous upregulation or downregulation of large-scale gene expression under specific conditions, rather than just the changes in the correlation of pairwise genes.

#### 2.3.2 Calculation of the hypergraph laplacian matrix

After constructing the hypergraph, the next step involves calculating the laplacian matrix of the hypergraph (Gao et al., 2020), which is used for further topological analysis. The hypergraph laplacian matrix provides a mathematical representation of the complex associations between nodes (genes) and hyperedges (samples) within the hypergraph, allowing for the capture of higher-order relationships among multiple genes and samples. The Laplacian matrix of the hypergraph is shown in Formula 2:

$$\Delta =D_{v}-HWD_{e}^{-1}H^{T}$$ (2)

where $D_{v}$ is the diagonal matrix of hyperdegrees of the gene nodes, $D_{e}$ is the diagonal matrix of hyperdegrees of the sample hyperedges, W is the weight matrix of hyperedges, and H is the incidence matrix of the hypergraph. The symbols appearing later have the same meanings as defined here. To further adapt to the scale of the data and ensure computational stability, the Laplacian matrix is normalized as Formula 3:

$$\Delta =I-D_{v}^{-1/2}HWD_{e}^{-1}H^{T}D_{v}^{-1/2}$$ (3)

here, I is the identity matrix. Compared to the laplacian matrix of simple graphs, this definition better reflects the higher-order structure in the weighted gene co-expression hypernetwork.

#### 2.3.3 Calculation of the topological overlap matrix

Next, based on the hypergraph laplacian matrix, we calculate the Topological Overlap Matrix (TOM), which reflects the topological overlap between gene pairs and serves to measure their similarity. For genes i and j , the TOM is computed as Formula 4:

$$TOM\left(i,j\right)=\frac{\Delta_{ij}+\sum_{k}\Delta_{ik}\Delta_{kj}}{\min\left(d_{i},d_{j}\right)+1-\Delta_{ij}}$$ (4)

where $\Delta_{ij}$ represents the value between genes i and j in the hypergraph laplacian matrix, and $d_{i}$ and $d_{j}$ are the degrees of genes i and j , respectively, indicating their connections to other genes. The TOM matrix serves as a measure of similarity for each pair of genes, aiding in the identification of gene pairs that are co-expressed across multiple samples, ultimately contributing to module clustering analysis.

#### 2.3.4 Gene module clustering

Upon obtaining the TOM matrix, WGCHNA employs hierarchical clustering to identify gene modules. Specifically, the dissimilarity of the Topological Overlap Matrix (dissTOM) is computed, defined as Formula 5:

$$dissTOM\left(i,j\right)=1-TOM\left(i,j\right)$$ (5)

to enhance the accuracy of module identification, the algorithm employs a dynamic module merging strategy. By dynamically detecting similar modules and merging them, it ensures that the identified modules exhibit higher stability and biological relevance.

#### 2.3.5 Module output and subsequent analysis

Ultimately, WGCHNA outputs the identified gene co-expression modules. These modules represent groups of co-expressed genes across different samples and can reflect potential biological functions or regulatory mechanisms. The identified gene modules can be further utilized for functional enrichment analysis, disease relevance studies, or other subsequent biological analyses.

### 2.4 Comparative experiments

To evaluate the effectiveness of WGCHNA, we compared it with traditional WGCNA methods [especially PyWGCNA(31) and R WGCNA(5)] and distance-correlation-based weighted gene co-expression network analysis [DC-WGCNA(18)]. WGCNA was originally widely used in the R programming language, but with the increasing influence of Python in the field of bioinformatics, the Python version of WGCNA has also been developed and gained widespread attention. Each version has unique advantages in terms of functions and application scenarios, suitable for different research needs. DC-WGCNA applies distance correlation to WGCNA. Compared with traditional WGCNA, DC-WGCNA can improve the results of enrichment analysis and the stability of the module. However, it has higher time complexity and requires more memory.

### 2.5 Experimental setup

To ensure a fair comparison of different methods under the same experimental conditions, we set strict and consistent hyperparameters for WGCHNA, PyWGCNA, R WGCNA, and DC-WGCNA to reduce the bias caused by parameter selection and ensure the comparability of experimental results. In the process of gene co-expression module detection, the minimum module value (minClusterSize) was uniformly set to 50 to ensure that the module contains a sufficient number of genes, thereby improving the stability of the module and avoiding the generation of too many small modules. At the same time, the module partition depth (deepSplit) was set to 2 to moderately control the granularity of module splitting, so that larger modules can be further decomposed while preventing excessive fragmentation. In addition, to improve the consistency of modules, we merged modules with a similarity of more than 80% to reduce module redundancy caused by random fluctuations and improve the biological relevance of modules.

## 3 Results and discussion

### 3.1 Data preprocessing and scale-free verification

The first step in WGCNA involves selecting an appropriate soft threshold to construct a gene co-expression network that exhibits scale-free properties. This step effectively captures the inherent characteristics of biological networks, specifically the uneven connectivity between a few hub genes and numerous other genes. When extending this analysis to a weighted hypergraph, it is similarly necessary to use a soft threshold to construct a weighted gene co-expression hypernetwork with scale-free properties. A scale-free hypernetwork not only captures the complex higher-order relationships among multiple genes but also ensures that the network’s topology aligns with the characteristics of real biological systems.

**FIGURE 4:**
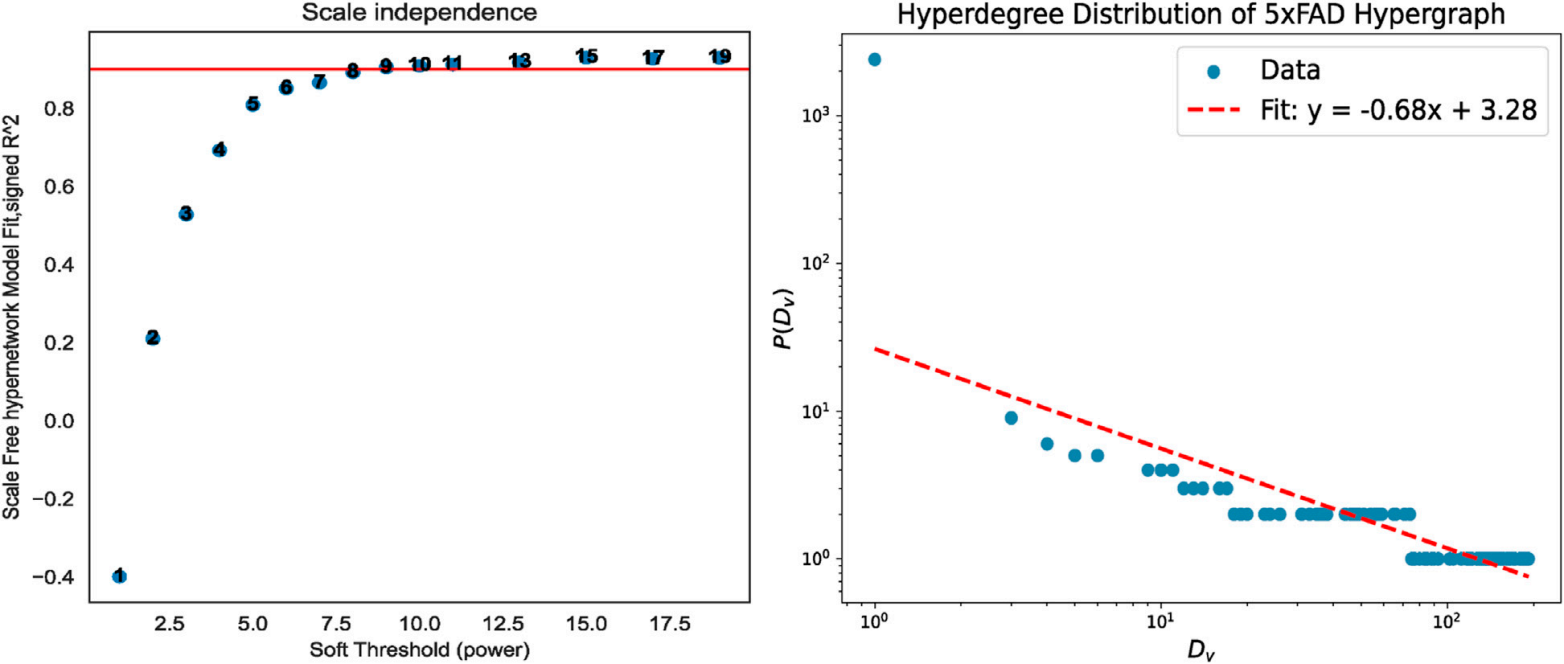
Soft threshold processing and scale-free validation. When the soft threshold is 9, the constructed hypernetwork conforms to the scale-free property.

Therefore, before the experiment begins, it is necessary to first perform missing value processing and soft threshold selection operations on each gene data set to construct a weighted gene co-expression hypernetwork. The hypernetwork constructed by the soft threshold we selected has been verified by the hyperdegree distribution law and all conforms to the scale-free feature. Take the 5xFAD data as an example, as shown in Figure 4, and other data results can be found in Supplementary Material 1.

### 3.2 Clustering performance evaluation

In this study, Silhouette Index, (SI) (Dudek, 2020), Calinski-Harabasz Index, (CHI) (Lima and Cruz, 2020), and Davies-Bouldin Index, (DBI) (Xiao et al., 2017) were used as internal clustering evaluation metrics to assess the quality of the clusters produced by various methods. Additionally, Adjusted Mutual Information, (AMI) (Lazarenko and Bonald, 2021) and Adjusted Rand Index, (ARI) (Chacón and Rastrojo, 2023) were employed as external clustering evaluation metrics to further evaluate the clustering quality of the datasets. The experimental results are listed in Tables 2, 3 respectively.

**TABLE 2:** Comparison of internal evaluation metrics obtained by WGCHNA with different modeling methods on all gene expression datasets.

Dataset	Evaluation measures	PyWGCNA	R WGCNA	DC-WGCNA	WGCHNA
5xFAD	SI	0.386	0.394	0.374	0.470
	CHI	2304.095	2237.917	2126.021	4909.988
	DBI ↓	0.807	0.861	0.872	0.680
GSE48213	SI	0.389	0.301	0.285	0.393
	CHI	4907.275	2835.172	2693.413	5132.688
	DBI ↓	0.870	0.992	0.996	0.830
GSE75360	SI	0.478	0.322	0.305	0.481
	CHI	1295.324	591.379	561.810	1304.344
	DBI ↓	0.691	0.928	0.931	0.681
GSE75670	SI	0.382	0.314	0.298	0.383
	CHI	2874.955	1949.226	1851.765	2888.122
	DBI ↓	0.834	0.898	0.903	0.831

**TABLE 3:** Comparison of external evaluation metrics obtained by WGCHNA with different modeling methods on all gene expression datasets.

Dataset	Evaluation measures	PyWGCNA	R WGCNA	DC-WGCNA	WGCHNA
5xFAD	AMI	0.287	0.277	0.263	0.334
	ARI	0.196	0.184	0.174	0.205
GSE48213	AMI	0.322	0.308	0.292	0.376
	ARI	0.214	0.218	0.207	0.231
GSE75360	AMI	0.196	0.195	0.185	0.201
	ARI	0.199	0.198	0.188	0.199
GSE75670	AMI	0.200	0.202	0.191	0.202
	ARI	0.200	0.200	0.190	0.201

The experimental data in Tables 2, 3 indicate that WGCHNA exhibits superior clustering performance on multi-sample datasets, especially in capturing complex gene expression patterns. However, the performance differences among methods become less pronounced when dealing with smaller or highly similar datasets.

#### 3.2.1 Performance advantages on multi-sample datasets

From the results in Tables 2, 3, WGCHNA demonstrates a clear advantage on larger datasets (such as 5xFAD and GSE48213), particularly in terms of internal evaluation metrics (SI, CHI, DBI) and external evaluation metrics (AMI, ARI).

• **5xFAD Dataset:** The SI of WGCHNA is 0.470, which is significantly higher than PyWGCNA (0.386), R WGCNA (0.394) and DC-WGCNA (0.374), indicating that WGCHNA is superior in the clarity of module boundaries and internal tightness. In terms of CHI index, WGCHNA achieved 4909.988, far exceeding other methods (PyWGCNA: 2304.095, R WGCNA: 2237.917, DC-WGCNA: 2126.021), indicating that it has a higher ability to distinguish differences between modules. For the DBI index, WGCHNA obtained a lower value of 0.680, while DC-WGCNA obtained a value of 0.872, further proving that WGCHNA has more advantages in maintaining internal consistency of modules. The AMI and ARI of WGCHNA are 0.334 and 0.205, respectively, which are higher than PyWGCNA (0.287 and 0.196), R WGCNA (0.277 and 0.184) and DC-WGCNA (0.263 and 0.174), indicating that WGCHNA is more accurate in reconstructing real biological modules.
• **GSE48213 Dataset:** The SI of WGCHNA is 0.393, which is slightly better than PyWGCNA’s 0.389, and significantly higher than R WGCNA (0.301) and DC-WGCNA (0.285). In terms of CHI value, WGCHNA achieved 5132.688, which is higher than PyWGCNA (4907.275), R WGCNA (2835.172) and DC-WGCNA (2693.413). In the DBI index, WGCHNA is 0.830, showing a higher intra-module compactness compared with DC-WGCNA’s 0.994. The AMI and ARI of WGCHNA are 0.376 and 0.231, respectively, which are higher than other methods, among which DC-WGCNA is 0.292 and 0.207, respectively, indicating that WGCHNA performs better in capturing complex gene expression patterns.

#### 3.2.2 Performance on small-scale or similar datasets

In contrast, on smaller or more homogeneous datasets, the performance differences among the methods are relatively minor. For instance, on the GSE75360 and GSE75670 datasets, the clustering results are more comparable across methods.

• **GSE75360 Dataset:** The SI of WGCHNA is 0.481, which is close to that of PyWGCNA (0.478), but significantly higher than R WGCNA (0.322) and DC-WGCNA (0.305). In the CHI index, WGCHNA (1304.344) is slightly better than PyWGCNA (1295.324), while DC-WGCNA is only 561.810. In DBI, WGCHNA (0.681) is lower than PyWGCNA (0.691), while DC-WGCNA’s 0.931 indicates poor consistency within the module. The differences in AMI and ARI among the methods are small, 0.201 and 0.199 for WGCHNA, respectively, while those for DC-WGCNA are 0.185 and 0.188, indicating that the methods perform relatively closely on smaller datasets.
• **GSE75670 Dataset:** The SI of WGCHNA is 0.383, which is basically the same as PyWGCNA (0.382), and both are better than R WGCNA (0.314) and DC-WGCNA (0.298). In terms of CHI value, WGCHNA (2888.122) is slightly higher than PyWGCNA (2874.955), while R WGCNA and DC-WGCNA are 1949.226 and 1851.765 respectively. In the DBI index, WGCHNA (0.831) is close to PyWGCNA (0.834), while DC-WGCNA achieves 0.913, indicating that the internal consistency of its clustering module is weak. In terms of AMI and ARI, WGCHNA is 0.202 and 0.201 respectively, slightly higher than DC-WGCNA’s 0.191 and 0.190, but the overall difference is small, reflecting that when the data scale is small or the features are simple, the performance of each method tends to be consistent.

In summary, WGCHNA showed significant advantages on multi-sample, large-scale gene expression datasets, and its performance in both internal and external indicators was better than other methods, including PyWGCNA, R WGCNA, and DC-WGCNA. In particular, WGCHNA showed higher accuracy and module consistency in capturing complex gene expression patterns and distinguishing differences between modules. In contrast, DC-WGCNA performed relatively poorly on all datasets, and its performance lagged behind WGCHNA in terms of clarity of module division, differences between modules, and internal tightness. For small-scale or similar-featured datasets, the performance differences between the methods were small, but in large-scale and complex data, WGCHNA was selected to more accurately reflect biological modules, providing more reliable support for subsequent functional annotation and mechanism studies.

### 3.3 Time complexity comparison

**FIGURE 5:**
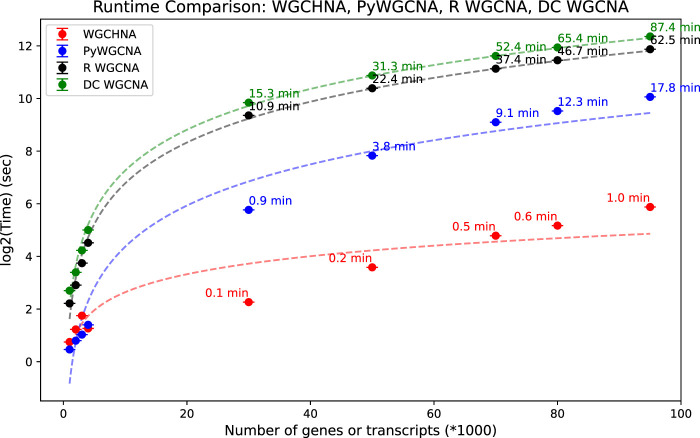
Comparison of running time of WGCHNA and Python and R versions WGCNA and DCWGCNA under different numbers of genes.

In the experiment, the average running time of four algorithms (WGCHNA, PyWGCNA, R WGCNA, DC-WGCNA) was calculated for gene datasets of different orders of magnitude, and the results are shown in Figure 5. The experiment covers gene datasets from small to large scales, aiming to evaluate the performance of each algorithm when processing different numbers of genes.

As can be seen from Figure 5, as the number of genes increases, the running time of the four algorithms increases, but there are significant differences in the magnitude and speed of the increase. When the amount of gene data is small, the running time of the four algorithms is not significantly different, and the performance of PyWGCNA, R WGCNA and DC-WGCNA is close to WGCHNA. However, as the scale of gene data increases, WGCHNA gradually shows a significant computational advantage, and its running time is significantly shorter than that of the other three algorithms, especially on large-scale data sets of thousands to tens of thousands of genes. Compared with the traditional WGCNA method (both Python and R versions) and DC-WGCNA, WGCHNA shows higher computational efficiency when processing more complex gene expression networks. Its advantage mainly comes from the use of a hypergraph structure in the algorithm design, which can effectively capture the high-order correlation between multiple genes, while reducing unnecessary pairwise correlation calculations, thereby reducing the overall computational complexity. On the other hand, as the number of genes increases, the running time of R WGCNA shows a more significant linear growth, and the computational overhead is higher on large-scale data sets, which may be related to the computational efficiency limitations of the R language itself. PyWGCNA has optimized the computational process to a certain extent. Although the performance gap between PyWGCNA and WGCHNA is not large on small and medium-sized data sets, the scalability of PyWGCNA is still inferior to WGCHNA on large-scale data sets. DC-WGCNA has a similar running time to R WGCNA on small-scale data, but also shows a significant time growth trend on large-scale data sets, and even the overall time consumption exceeds R WGCNA, indicating that its algorithm scalability still needs to be further optimized. In general, WGCHNA can maintain high computational efficiency on data sets of different sizes, especially showing more outstanding performance advantages in large-scale data analysis scenarios.

### 3.4 Identification of co-expression modules

**FIGURE 6:**
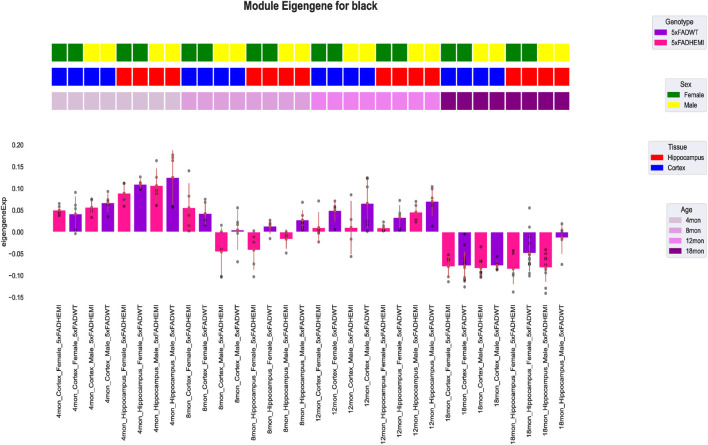
Visualization of the correlation between the black module characteristic gene expression profile module and sample traits in the 5xFAD mouse model. In the figure, the first three rows show the traits of each sample, including sex, tissue, and age. The bar graph shows the module characteristic gene expression of each sample divided by genotype, and the module characteristic gene expression of a single sample is shown as a point.

In the subsequent analysis, WGCHNA used the same method as WGCNA to construct gene co-expression modules. Specifically, we calculated the topological overlap matrix (TOM) based on the hypergraph Laplacian matrix and used the dynamic tree cutting method to identify gene co-expression modules. Figure 6 shows the 5xFAD gene modules identified by WGCHNA and their associations with sample traits, of which only the black modules are shown. The detailed results of other modules can be found in Supplementary Material 2. The selection of modules was based on the following criteria: First, the selected modules achieved high statistical significance (p < 0.05) in the correlation analysis with sample traits, indicating that they may have important biological significance in the 5xFAD model. Second, the modules contained multiple core genes (Top-ranked genes) with high connectivity, which played a key role in the co-expression network. In addition, functional enrichment analysis showed that the selected modules were significantly associated with biological processes such as Alzheimer’s disease-related pathways or neuroinflammatory responses. Finally, the modules showed high robustness under different parameter settings, further verifying their structural reliability and biological explanatory power. Therefore, we highlighted the black modules as representative cases to illustrate the effectiveness and biological explanatory power of WGCHNA in gene module identification.

As shown in Figure 6, the expression patterns of two gene modules (black) in the 5xFAD dataset vary under different experimental conditions. The color bars represent sample genotype, sex, tissue type, and age group. The expression of genes in the black module exhibits significant variation across conditions, particularly at specific ages (e.g., 8 and 12 months) and in hippocampal tissues, where 5xFAD/WT genotype samples show higher characteristic gene values.

### 3.5 Downstream analysis of co-expression modules

**FIGURE 7:**
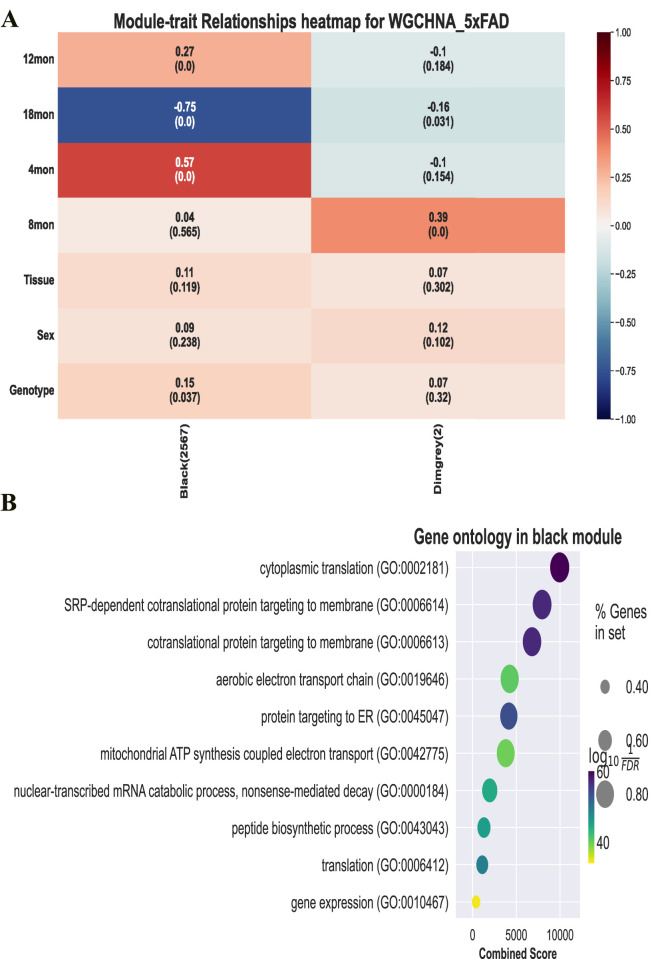
Downstream analysis and visualization of co-expression modules. **(A)** Module-trait correlation calculation. **(B)** GO function enrichment analysis of black modules.

WGCHNA supports the same downstream analysis functions and visualization of co-expression modules as PyWGCNA. It can calculate module-trait correlations and summarize the expression of module characteristic genes in sample source data (Virshup et al., 2021; Fang et al., 2023), detect hub genes in each module, and perform functional enrichment analysis in each module using databases such as GO, KEGG, and REACTOME (Ashburner et al., 2000; Fabregat et al., 2018; Szklarczyk et al., 2021). Figure 7 shows the functional enrichment analysis results of the black module in the 5xFAD gene co-expression module identified by WGCHNA. Other related results can be found in Supplementary Material 3.

Figure 7 presents the trait correlations and functional annotations of genes in the black module identified by WGCHNA. The heatmap(A) shows the correlation between the module eigengenes and various traits (e.g., age, sex), revealing a significant negative correlation with samples at 12 months of age and a positive correlation at 18 months. The bubble plot(B) highlights the primary biological processes involving the module genes through GO functional annotation, including cytoplasmic translation, protein targeting to membranes, and mitochondrial ATP synthesis.

### 3.6 Literature verification and enrichment analysis

**FIGURE 8:**
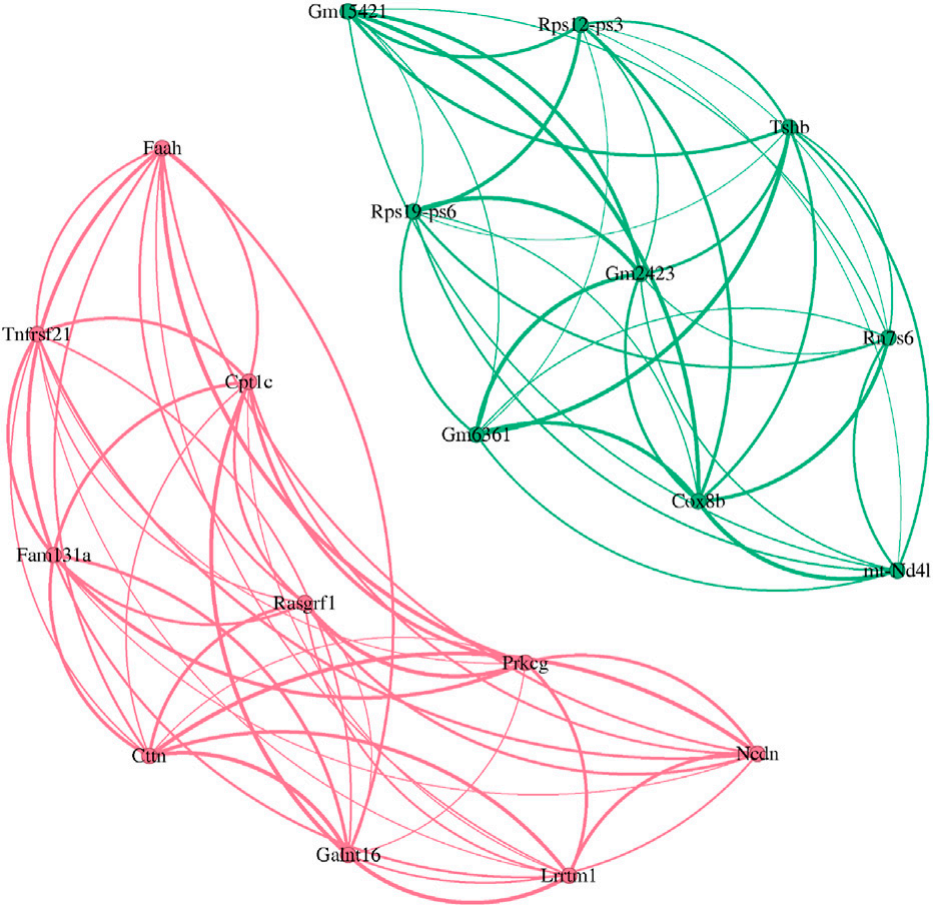
Comparison of top-10 gene module recognition. (Pink) WGCHNA (Green) PyWGCNA.

Existing studies have shown that hypergraph network modeling methods (Feng et al., 2021; Tian et al., 2009; Barton et al., 2023) have significant advantages in complex biological network analysis, especially in the identification of key gene modules. For example, the study by Tian et al. (Feng et al., 2021) showed that by introducing a hypergraph model, the complex synergy between multiple genes can be more effectively captured. In particular, when processing multi-sample disease data, the hypergraph model can screen out gene modules that are highly related to the disease. To verify this point of view, this paper takes the mouse Alzheimer’s disease dataset (5xFAD) as an example to compare the performance of the two methods WGCHNA and PyWGCNA. Table 4 shows the top 10 genes in the gene co-expression modules mined by the two methods. N/A means that the relevant genes could not be identified due to the limitation of the numerical calculation accuracy of the algorithm. To intuitively demonstrate this phenomenon, we added a visualization diagram of the WGCHNA and WGCNA recognition modules, as shown in Figure 8.

**TABLE 4:** Comparison of 5xFAD gene co-expression modules.

Algorithm	Top-10				
PyWGCNA	Cttn	Prkcg	Lrrtm1	Ncdn	Rasgrf1
	Fam131a	Tnfrsf21	Faah	Galnt16	Cpt1c
WGCHNA	Rps12-ps3	N/A	Tshb	mt-Nd4l	Gm15421
	Gm6361	Rn7s6	Rps19-ps6	Cox8b	Gm2423

Comparative analysis reveals significant differences between PyWGCNA and WGCHNA in identifying key genes. PyWGCNA identifies the top 10 genes including Cttn (Sarabi et al., 2024), Prkcg (Jiang et al., 2019), and Rasgrf1(50) (Song et al., 2016), which are previously reported to be associated with neurodegenerative diseases such as Alzheimer’s disease. However, PyWGCNA encounters substantial noise interference in certain gene detections (e.g., Cttn, Prkcg), potentially introducing uncertainties in the functional information of these genes (Sarabi et al., 2024; Jiang et al., 2019).

In contrast, WGCHNA identifies a more diverse set of gene modules, capturing genes associated with crucial biological processes such as mitochondrial function and translation regulation. For instance, mt-Nd4l (Zhang et al., 2022) and Cox8b (Xiyang et al., 2020), mitochondrial DNA-encoded genes known to be closely related to neuronal energy metabolism, where energy metabolism dysfunction is a major pathological mechanism of Alzheimer’s disease. This suggests that WGCHNA can more effectively capture complex higher-order gene interactions highly relevant to disease mechanisms. Although WGCHNA encounters cases of N/A in specific gene identifications, possibly due to algorithmic precision limitations under certain computational conditions, this does not significantly affect the overall biological functionality of the modules. Overall, WGCHNA identifies a more biologically relevant list of genes, particularly excelling in mining disease-related modules.

As shown in Figure 8, the pink part represents the gene module identified by WGCHNA, while the green part comes from the analysis results of WGCNA. It can be seen that the pink module identified by WGCHNA establishes denser connections between multiple genes, indicating that under the hypergraph model, these genes frequently co-occur in multiple samples (hyperedges), forming functional modules with higher-order co-expression characteristics. In contrast, the green module identified by WGCNA mainly relies on pairwise correlation metrics, and the presented network structure emphasizes the linear relationship between genes, which makes it difficult to reflect the overall effect of multi-gene joint expression. By comparing the gene composition and functional annotations of the two modules, we can further verify the advantages of WGCHNA in mining complex co-expression relationships.

**FIGURE 9:**
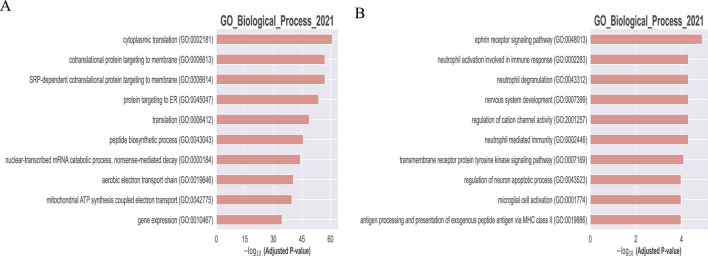
Comparison of GO enrichment analysis of Top-10 genes. **(A)** WGCHNA **(B)** PyWGCNA.

To further validate the differences between the two methods, we conducted GO enrichment analysis on the identified gene modules. Figure 9 illustrates the enrichment results, where panel A represents the enrichment analysis by WGCHNA and panel B by PyWGCNA.

Go functional enrichment analysis highlights three aspects demonstrating the superiority of WGCHNA: Firstly, gene modules identified by WGCHNA exhibit higher statistical significance, particularly in pathways closely associated with disease core mechanisms such as mitochondrial function and energy metabolism. Secondly, WGCHNA reveals a more diverse spectrum of biological processes, encompassing multiple levels of biological functionality including translation, protein targeting, and gene expression regulation, whereas PyWGCNA’s enrichment results are relatively focused on limited biological processes. Finally, WGCHNA captures complex gene interactions through higher-order modeling, demonstrating stronger analytical capabilities, and thus exhibits significant advantages in identifying critical gene modules relevant to diseases.

### 3.7 The role of hyperedge weights in capturing high-order co-expression patterns

**FIGURE 10:**
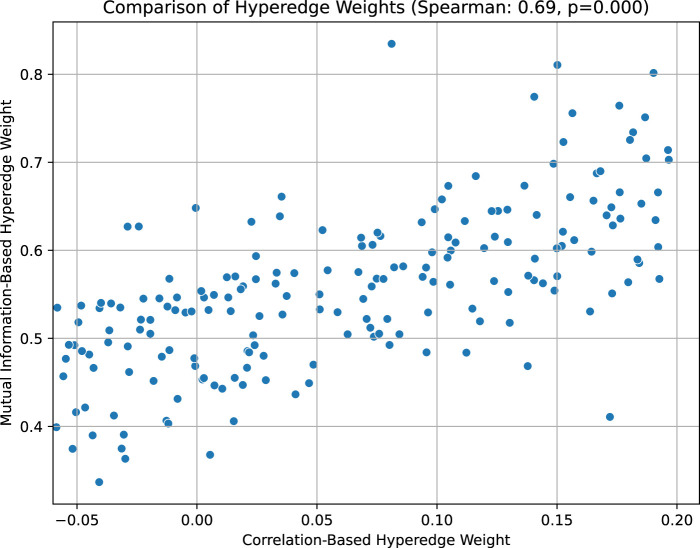
Comparison of hyperedge weights based on correlation and mutual information in the 5xFAD dataset.

In this method, we construct hyperedges based on samples, one hyperedge for each sample, connecting all genes in the sample. This design can effectively retain high-order information about the overall gene expression in the sample. For example, under a certain condition, even if 20 of 40 genes are significantly upregulated and 20 are significantly downregulated, but the pairwise correlation between the genes is close to zero, the hyperedge construction still ensures that all upregulated genes and downregulated genes co-occur in the same hyperedge. The hyperedge weights can reflect the expression trends in the sample as a whole by aggregating the correlations of all gene pairs in the sample, rather than relying solely on individual pairwise relationships. This aggregation effect allows the overall co-expression pattern to be accurately captured even if the local pairwise correlation is not significant. In addition, the hyperedge weights provide an important basis for the subsequent division of gene co-expression modules. The close association between the hyperedge weights and the multi-gene joint expression patterns is further demonstrated by statistical methods, thereby improving the biological significance and explanatory power of the constructed modules. Taking the 5xFAD dataset as an example, we calculate the hyperedge weights using the correlation-based and mutual information-based methods respectively, and compare them in the Figure 10 (as shown in the Figure 10, the horizontal axis is the hyperedge weight measured by correlation, and the vertical axis is the hyperedge weight measured by mutual information).

The Figure 10 above shows the relationship between the “hyperedge weight based on correlation” (horizontal axis) and the “hyperedge weight based on mutual information” (vertical axis) of 192 hyperedges. Each scatter point represents a hyperedge composed of several genes, and its horizontal axis value is the average correlation of all gene pairs in the hyperedge, and the vertical axis value is the average mutual information of all gene pairs in the hyperedge. It can be seen that most of the scatter points are distributed from the lower left to the upper right, showing an obvious positive correlation trend. By calculating the Spearman correlation coefficient (Spearman = 0.69, p = 0.000), we found that there is a moderately strong positive correlation between the two weights, and it is statistically significant. This shows that when the genes of a hyperedge are more closely related in pairwise correlation, they tend to show stronger synergistic expression in higher-order mutual information metrics, thus supporting the hypothesis that hyperedge weights can effectively reflect the joint action of multiple genes.

## 4 Conclusion

This study introduces Weighted Gene Co-expression Hypernetwork Analysis (WGCHNA), a novel approach grounded in hypergraph theory, designed to overcome the limitations of traditional WGCNA in capturing pairwise gene interactions. By constructing a weighted hypergraph, WGCHNA integrates high-order gene interactions, utilizing the hypergraph Laplacian matrix to characterize complex co-expression patterns with greater precision. This enables the identification of co-expression modules that are closely associated with key biological processes.

Experimental evaluations demonstrate that WGCHNA significantly outperforms both the Python and R implementations of WGCNA and DCWGCNA in computational efficiency, particularly in handling large-scale datasets with multiple samples and intricate gene interaction networks. Moreover, the hypergraph-based modeling approach enhances the ability to dissect complex gene interactions while achieving substantial improvements in computational performance. Notably, WGCHNA excels in pathogenic gene screening, successfully identifying key genes linked to Alzheimer’s disease (e.g., mt-Nd4l and Cox8b). These findings not only confirm previously established biological knowledge but also reveal novel regulatory relationships, further substantiating the crucial roles of these genes in neuronal energy metabolism.

In terms of functional enrichment analysis, WGCHNA effectively identifies a diverse range of biologically relevant gene modules, encompassing pathways related to energy metabolism, mitochondrial function, protein targeting, and gene expression regulation, demonstrating its broad applicability in disease mechanism research and biological discovery.

Looking ahead, with advancements in big data analytics, the computational efficiency and module detection capabilities of WGCHNA are expected to improve, facilitating the analysis of even larger-scale and more complex gene interaction networks. Additionally, integrating WGCHNA with high-order network modeling frameworks such as directed hypergraphs and simplicial complexes will enable deeper exploration of nonlinear and high-order genetic interactions, potentially unveiling novel gene regulatory mechanisms that remain undiscovered.

## Data Availability

The data presented in this study are openly available in https://www.model-ad.org/}[MODEL-AD], and https://www.ncbi.nlm.nih.gov/}[NCBI]. The Python code associated with this research has been released in https://zenodo.org/records/15023675}[Zenodo].
