# Supplementary material for: Hypergraph-based analysis of weighted gene co-expression hypernetwork
Source: Front Genet. 2025 Apr 4;16:1560841. doi: 10.3389/fgene.2025.1560841 (PMC12006133; doi:10.3389/fgene.2025.1560841)
Supplement: Supplementary file 1 [file DataSheet1.pdf]

Supplementary Material

1 SUPPLEMENTARY FIGURES 1: SOFT THRESHOLD PROCESSING AND SCALE-FREE VALIDATION

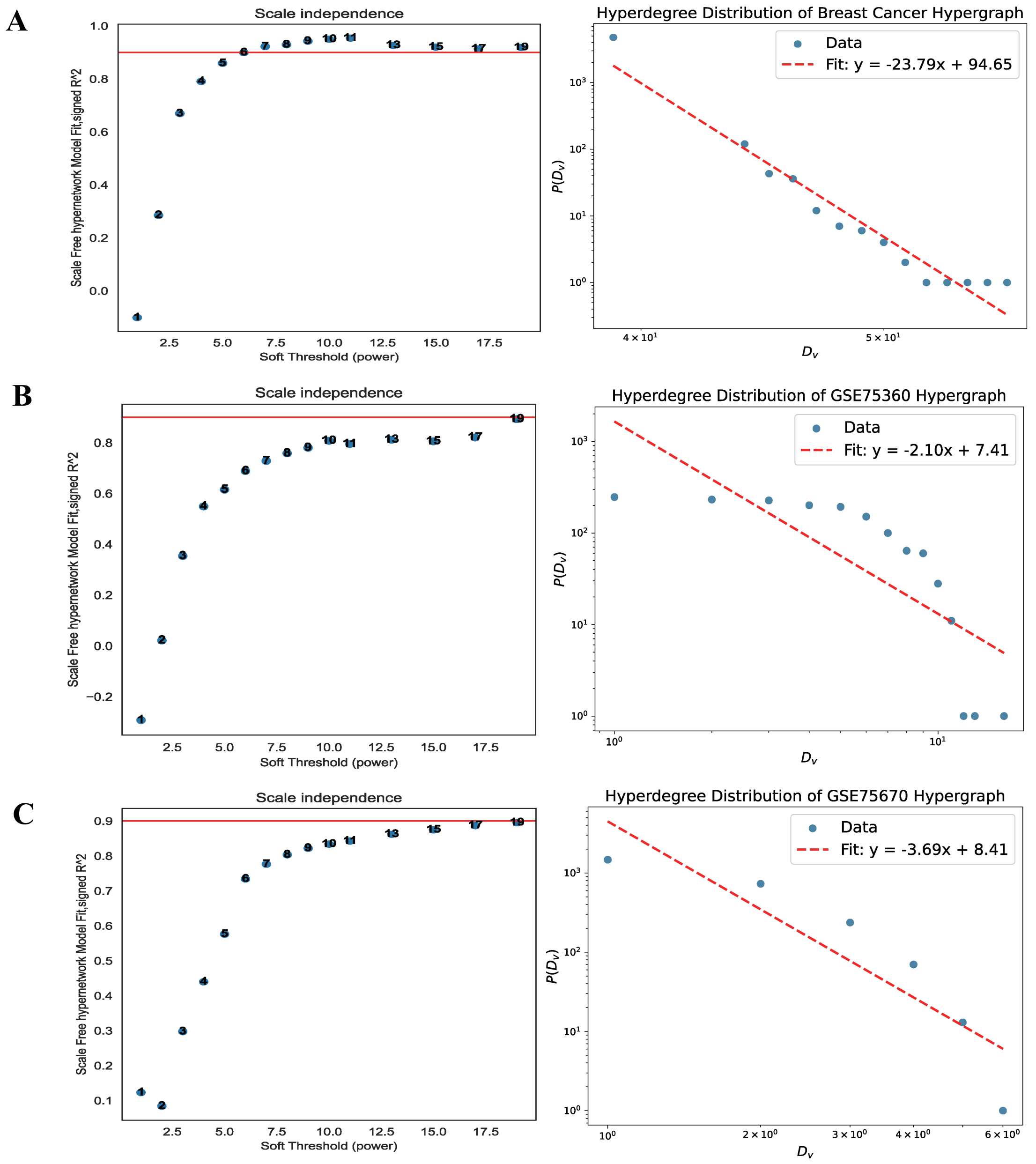

Figure S1. Soft threshold processing and scale-free validation (A) is the GSE48213 dataset. The soft threshold is 6. (B) and (C) are the GSE75360 and GSE75670 datasets. The soft threshold is 19.

2 SUPPLEMENTARY FIGURES 2: VISUALIZATION OF THE CORRELATION BETWEEN THE DARKGREY MODULE CHARACTERISTIC GENE EXPRESSION PROFILE MODULES AND SAMPLE TRAITS IN THE 5XFAD MOUSE MODEL

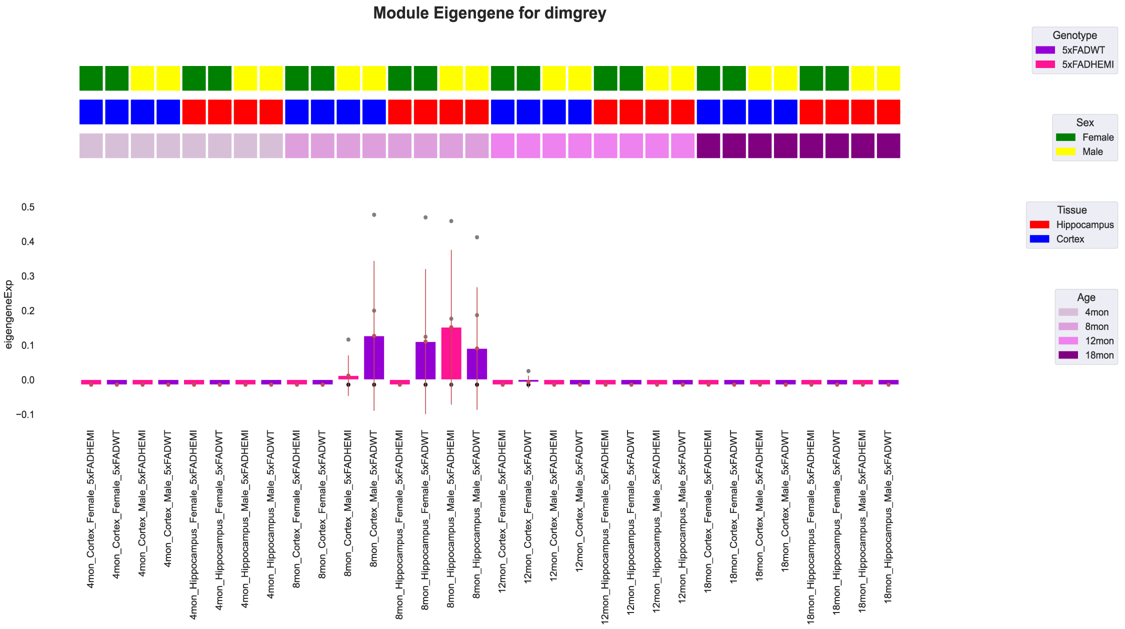

Figure S2. Visualization of the correlation between the darkgrey module characteristic gene expression profile modules and sample traits in the 5xFAD mouse model

The gene expression of the dark gray module in 5xFAD was relatively low and did not change much under most conditions, except for some increased trends in the 5xFAD/HEMI group at 8 months old.

3 SUPPLEMENTARY FIGURES 3: HUB GENES AND THEIR PROTEIN-PROTEIN INTERACTION DIAGRAM

4 SUPPLEMENTARY 4: DATA AVAILABILITY STATEMENT

The data presented in this study are openly available in [MODEL-AD] , and [NCBI].The Python code associated with this research has been released in [Zenodo].

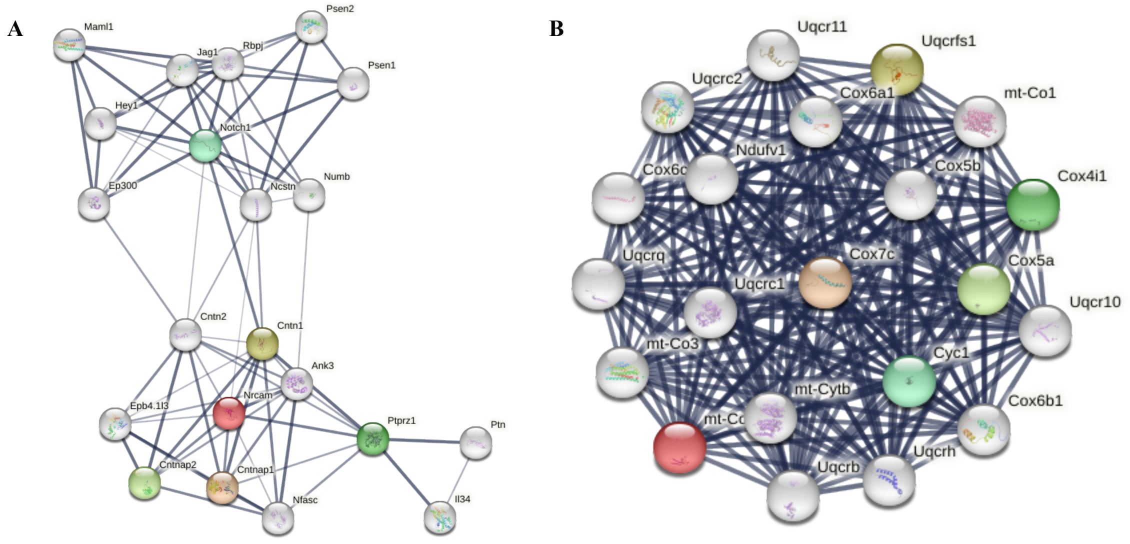

Figure S3. Protein-protein interaction graph of hub genes in the module identified by the WGCHNA algorithm. (A)cntn1_PPI (B) Cox5a_PPI
